# Environmental cell for *in situ* X-ray synchrotron micro-CT imaging with simultaneous acoustic measurements

**DOI:** 10.1107/S1600577521013308

**Published:** 2022-01-27

**Authors:** Arkady N. Drobchik, Viktor V. Nikitin, Mikhail I. Fokin, Geser A. Dugarov, Pavel D. Shevchenko, Alex L. Deriy, Andrey Yu. Manakov, Konstantin E. Kuper, Anton A. Duchkov

**Affiliations:** a Institute of Petroleum Geology and Geophysics SB RAS, 630090 Novosibirsk, Russia; b Advanced Photon Source, Argonne National Laboratory, Lemont, IL 60439, USA; c Nikolaev Institute of Inorganic Chemistry SB RAS, 630090 Novosibirsk, Russia; d Budker Institute of Nuclear Physics, 630090 Novosibirsk, Russia; e Novosibirsk State Technical University, 630087 Novosibirsk, Russia

**Keywords:** micro-tomography, environmental cell, acoustics, gas hydrates

## Abstract

Synchrotron radiation provides the necessary spatial and temporal resolution for non-invasive operando studies of dynamic processes under complex environmental conditions. Here a new environmental cell for simultaneous *in situ* dynamic X-ray imaging and measuring acoustic properties of geological samples is presented. The primary purpose of this cell is to study gas-hydrate formation in porous geo-materials and its influence on their acoustic properties. The cell is designed for cylindrical samples of 9 mm in diameter, confining and pore pressures up to 12 MPa, and temperatures from −20°C to room temperature. The cell is portable and can be easily assembled and operated at different X-ray sources. This cell enables a wide range of experiments studying physical/chemical processes in the Earth subsurface that change the mechanical properties of rocks (geochemical reactions, phase transitions, *etc*.).

## Abstract 2

A new environmental cell for simultaneous *in situ* dynamic X-ray imaging and measuring acoustic properties of geological samples is presented. The cell helps studying physical/chemical processes in geomaterials and their influence on the mechanical properties.

## 1. Introduction

Geological subsurface processes are complicated as they are multi-scale and multi-physical in nature. Laboratory petrophysical experiments are designed to reproduce *in situ* conditions and study how they affect the physical properties of rock samples (Tiab & Donaldson, 2015 ▸). X-ray micro-computed tomography (micro-CT) provides a powerful tool for *in situ* 3D imaging of geomaterials during petrophysical studies. Microtomography imaging data can be used for further analysis and upscaling of the petrophysical properties from the sample to the reservoir scale (Liu *et al.*, 2016 ▸). While laboratory X-ray sources can be used for reconstructing high-resolution volumes of a sample micro-structure, synchrotron-based imaging is much faster (Maire & Withers, 2014 ▸), making it possible to study dynamic processes with non-invasive operando imaging.

Recent years show rapid development in the field of synchrotron-based imaging of processes in geo-materials. The main advancement is the ability to perform dynamic (time-resolved) imaging of changes in the sample during experiments that mimic real geological processes. Fusseis *et al.* (2014 ▸) presented a low-cost X-ray-transparent environmental cell for imaging fluid–rock interactions under natural reservoir conditions (confining pressure up to 20 MPa, pore fluid pressure up to 15 MPa, temperature up to 200°C). There are cells for *in situ* micro-CT imaging of coupled processes of deformation and chemical reactions in rock samples: 3 mm in diameter, up to 50 MPa confining pressure, 600 MPa axial load (Butler *et al.*, 2020 ▸), 5 mm in diameter, up to 200 MPa confining pressure, 600 MPa axial load, 250°C temperature (Renard *et al.*, 2016 ▸). There is a triaxial cell for combined high-pressure (up to 24 MPa) and high-temperature (up to 400°C) *in situ* synchrotron micro-CT imaging of fluid flow experiments in geothermal applications (Voltolini *et al.*, 2019 ▸). Cai *et al.* (2014 ▸) presented a cell for cyclic loading of coal samples with simultaneous micro-CT imaging and measuring water permeability and acoustic emission (the P-wave velocity was measured separately after extracting the sample from the cell). Another environmental cell for *in situ* time-resolved imaging of a two-phase flow in a porous limestone sample during brine injection was presented by Bultreys *et al.* (2016 ▸).

Freezing and gas-hydrate formation considerably change the acoustic and mechanical properties of loose materials. Simultaneous micro-CT imaging and acoustic measurements may help to better understand how kinetics, thermodynamics, and the morphology of the hydrate/ice formation in pores affect the acoustic macro-properties of samples. Studying gas-hydrate formation/dissociation is of particular interest as natural gas hydrates are considered as a resource of natural gas, as well as a cause of climate change and geohazards (Koh & Sloan, 2007 ▸). Good knowledge of the physical properties of gas-hydrate-bearing sediments is important for further development of geophysical methods for gas-hydrate accumulation exploration (Riedel *et al.*, 2010 ▸). Specialized laboratory setups have been used for gas-hydrate formation in rock samples and studying various physical properties (Winters *et al.*, 2000 ▸; Schicks *et al.*, 2011 ▸). However, these setups are unable to perform dynamic imaging of the sample micro-structure and attribute variations in physical properties to changes in hydrate saturation, morphology or spatial distribution. This may cause difficulties in interpreting the experimental results and constructing effective models – see discussion by Dugarov *et al.* (2019 ▸).

Micro-CT imaging of the hydrate formation can be performed using laboratory X-ray sources that require several hours for scanning (Chen & Espinoza, 2018 ▸; Lei *et al.*, 2019 ▸). Synchrotron-based X-ray micro-CT imaging of xenon hydrate formation and dissociation of xenon hydrate was reported by Chaouachi *et al.* (2015 ▸) and Yang *et al.* (2016 ▸). A modified version of the environmental cell by Fusseis *et al.* (2014 ▸) was used for fast dynamic micro-CT imaging of methane-hydrate formation in sand and coal samples (Nikitin *et al.*, 2020 ▸, 2021 ▸). Usually one has to use separate experimental rigs: one for micro-CT imaging, the other for measuring physical properties (Schindler *et al.*, 2017 ▸; Falcon-Suarez *et al.*, 2020 ▸). Multimodal measurements in turn are rarely used in practice due to their complexity.

The need for simultaneous micro-CT imaging and acoustic measurements requires careful choice of the sample diameter. On the one hand, one wants to improve the imaging resolution to study processes in meso- and micro-pores. For example, Nikitin *et al.* (2020 ▸) used 1.725 µm resolution imaging to study gas-hydrate formation in pores of Ottawa fine white sand (grain size 125–250 µm); the sample diameter was 5 mm, the scanning region diameter was 4.2 mm. On the other hand, the diameter of the sample should be big enough to make successful acoustic measurements with piezo-ceramic transducers. For example, a sample of 2 cm in diameter was used for acoustic measurements during the hydrate formation in sand (Schindler & Batzle, 2015 ▸).

The main purpose of this paper is to present an environmental cell for simultaneous synchrotron-based micro-CT imaging and measuring acoustic properties (P-wave velocity). Our focus is to study how gas-hydrate formation affects the acoustic properties of sedimentary rocks. This information will be further used for developing seismic methods characterizing natural gas-hydrate accumulations and assessing their mechanical properties. While in this paper the cell is tested for forming gas-hydrates, it can also be employed in other studies such as geochemical reactions (*e.g.* in tailings dumps) and phase transitions that considerably change the mechanical properties of geological materials (*e.g.* permafrost degradation).

In this paper, we first describe the structure of the proposed environmental cell. Then we show an example of measuring acoustic properties and *in situ* micro-CT imaging of a sample during the process of gas-hydrate formation. We finish with a discussion and outlook.

## 2. Structure of the environmental cell

**Figure 1:**
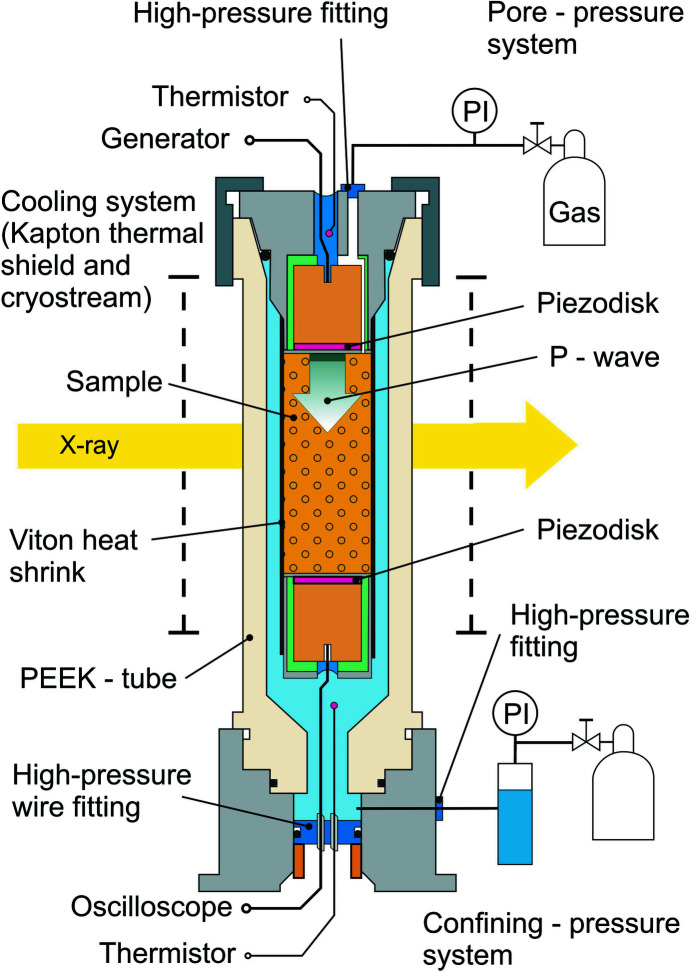
Schematic of the environmental cell for *in situ* dynamic X-ray imaging and measurement of acoustic properties (for technical drawings see the supporting information.

A schematic of the proposed environmental cell for micro-CT imaging and acoustic measurements is shown in Fig. 1 ▸. The sample is packed between two piezo-ceramic acoustic sensors connected by a Viton heat shrink tubing. The diameter of the packed cylindrical sample is 8.4 mm and the height is 10.5 mm. The upper acoustic sensor has a channel for pore pressure and gas supply to the sample. This sample pack is placed into the X-ray transparent polyether ether ketone (PEEK) pressure vessel with inner diameter of 11 mm and total height of 20 mm. Technical drawings for this environmental cell are provided in the supporting information.

High-pressure PEEK tubes for the confining oil and pore-pressure circuits are connected to the top and bottom parts of the cell using high-pressure fittings. These PEEK tubes allow the cell to rotate freely on the rotation stage over 180°. The lower acoustic receiver is connected to an oscilloscope via a high-pressure wire fitting. The upper acoustic transmitter is connected to a pulse generator. The wire connection for the upper transmitter does not require isolation from high gas and oil pressure. The present version of the cell has only P-wave transmitter and receiver. Signals from temperature sensors placed at the top and the bottom of the cell are measured through wires in the same cable.

**Figure 2:**
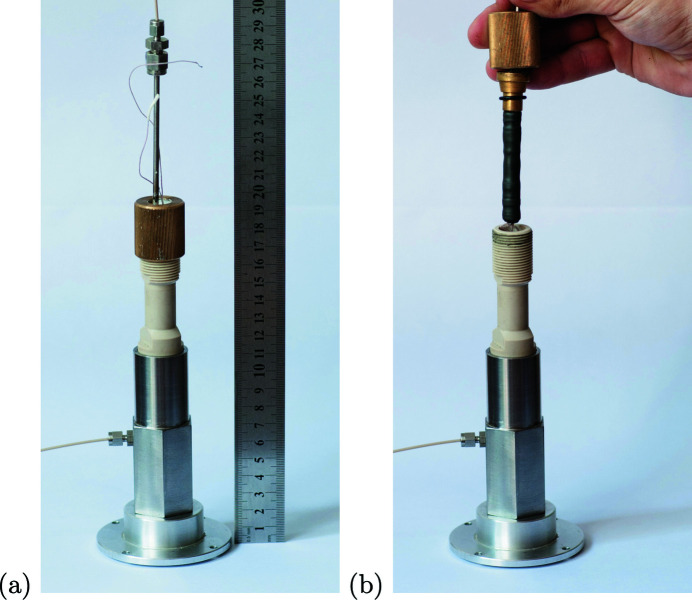
Experimental environmental cell for studying geo-materials: assembled cell (*a*), sample pack to be placed into the cell (*b*).

One can see the appearance of the cell in Fig. 2 ▸(*a*). At the bottom there is a high-pressure fitting for the confining pressure circuit, and at the top there is the fitting for gas supply and pore pressure. We first assemble the sample pack – the sample confined between two piezo-ceramic sensors and fixed by a Viton heat-shrink tubing. Then we place this sample pack into the confining pressure vessel, see Fig. 2 ▸(*b*). The cell is designed for a sample that is up to 20 mm long and 8–9 mm in diameter. Such a diameter still enables us to measure the velocity of body P-waves and perform 3D imaging of almost the whole sample – see discussion in the following subsections.

## 3. Application for studying gas-hydrate formation processes

The cell was tested at imaging beamlines from two synchrotron facilities: Advanced Photon Source (APS; Argonne National Laboratory, USA) and VEPP-3 (Budker Institute of Nuclear Physics, Russia). Here we show several examples from the cell testing.

### 3.1. Tomographic data acquisition and processing

**Figure 3:**
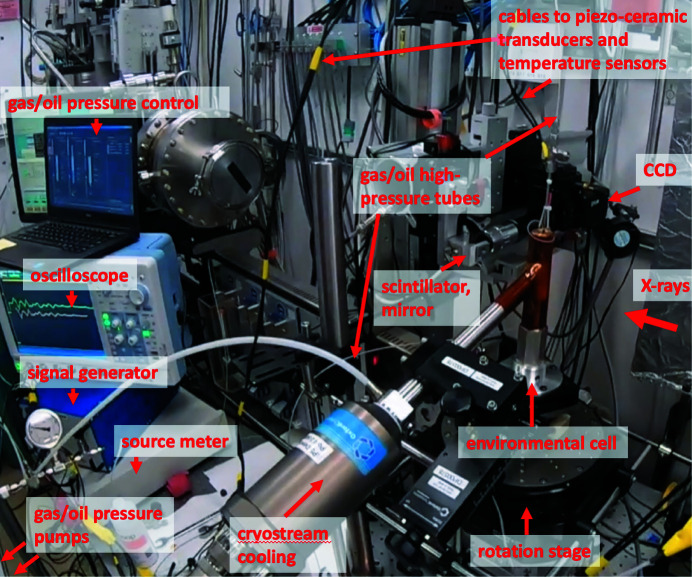
Experimental setup at Sector 2-BM (Advanced Photon Source) for micro-CT imaging of gas-hydrate formation in the environmental cell.

In Fig. 3 ▸ we show an example of the cell installation at Sector 2-BM (bending magnet) of the APS. This beamline allows flexible X-ray energy selection and adjustable optics for sub-10 µm pixel sizes, and is adapted for conducting *in situ* experiments of studying dynamic processes with complex instrument setups and sample environment conditions.

The left part of Fig. 3 ▸ shows the location of devices for controlling and measuring the pressure and temperature conditions and working with acoustic signals, which include a gas pressure pump (Teledyne ISCO D-1000 pump), an oil pressure pump (neMESYS Syringe Pumps), a source meter (Keithley 2612A), a signal generator (Siglent Technologies SDM3045X), an oscilloscope (Yokogawa DLM5000), and a cryostream cooling system (Oxford 700 Cryostream). Note that all these devices or their analogs are typically available upon request at synchrotron facilities. In the right part of Fig. 3 ▸ one can see the environmental cell placed on a rotation stage, high-pressure PEEK tubes and cables connected to the cell, and the beamline micro-CT acquisition system. The detector assembly comprises a 100 µm-thick LuAG:Ce scintillator, a 2× long-working-distance Mitutoyo objective lens, and a FLIR Oryx (model 10GS 51S5) CCD. The CCD chip is made of 2448 × 2048 pixels with 3.45 µm pixel size, which after 2× magnification yields 1.725 µm object voxel sizes and 4.2 mm × 3.5 mm field of view. Because of the limited beam size in the vertical direction, the field of view for data acquisition was decreased to 4.2 mm × 1.75 mm. Control of all aforementioned devices for working with the environmental cell was carried out remotely using the Experimental Physics and Industrial Control System (EPICS).

**Figure 4:**
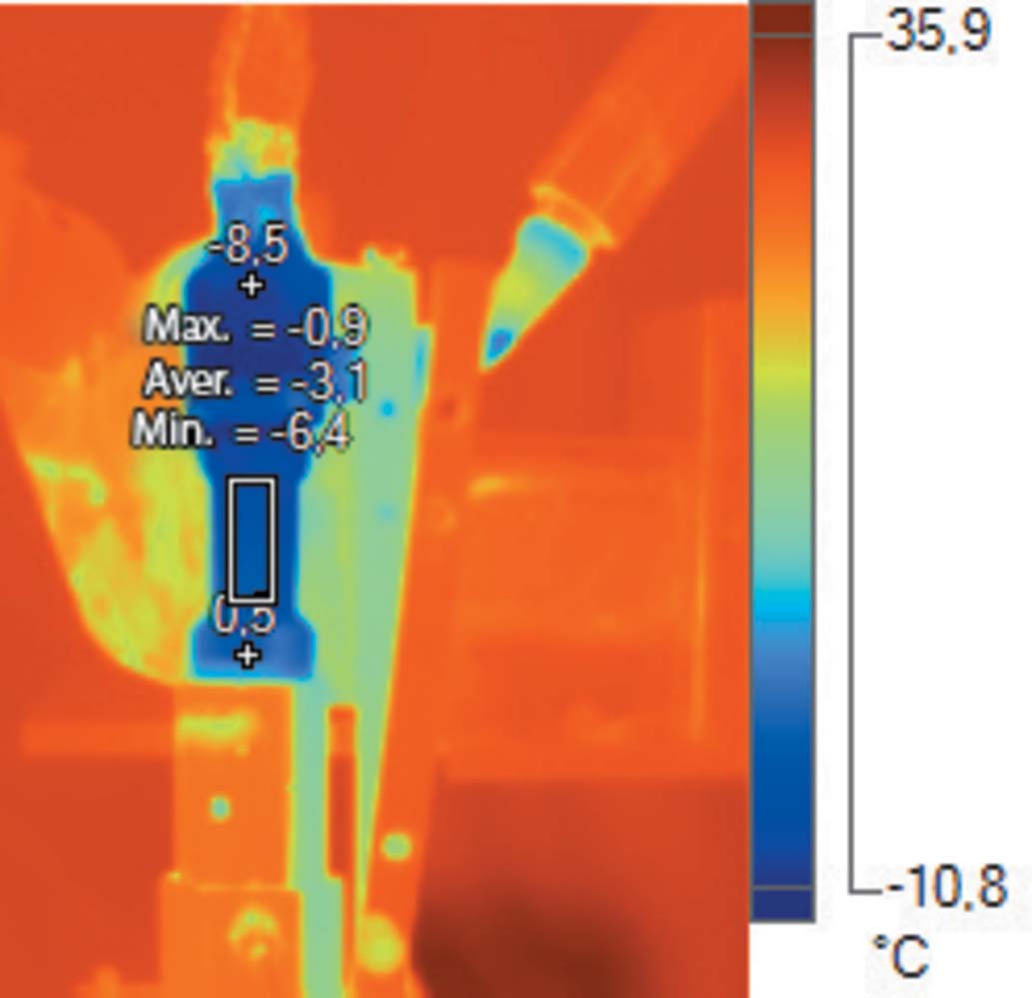
Thermal image of the cell during cryostream cooling.

For our experiment it was crucial to be able to cool down the cell below freezing. For cooling we used a cryostream system supplied with liquid nitrogen. A Kapton cylinder was placed to isolate the volume surrounding the side walls of the cell. The cryostream nozzle was placed into a Kapton sleeve at an angle such that it blows nitrogen not against the cell wall but tangentially. The aim here is to form a spiral nitrogen flow that turns around the cylindrical cell for uniform cooling of the walls. We took thermal images to check that cooling of the central part of the cell is indeed uniform, see Fig. 4 ▸. To ensure uniform cooling of the sample during the whole scanning period, the cryostream nozzle was mounted on the bottom part of the rotation stage and moved together with the environmental cell in the horizontal and vertical directions.

One of the goals in the gas-hydrate formation experiments was to estimate the total amount of different substances (water, gas, gas hydrate) inside the environmental cell at different time steps of the formation period. The statistically representable imaging area for this experiment was a cylinder with diameter 8.4 mm and height 10.5 mm, much larger than the detector field of view. In order to perform continuous scanning of the whole sample region in the horizontal direction we set the rotation axis at the border of the field of view and acquired data for 0°–360° angles with overlaps. The projections were then stitched and processed as a regular 180° scan with twice the number of pixels in the horizontal direction and reconstructed image diameter of 8.4 mm. To perform a full vertical scan of the sample, we acquired data at vertically translated positions. Scanning for different positions was performed with a small overlap for accurate data stitching.

The incident X-ray beam was formed after (1) carbon filtering to cut photon energies lower than 15 keV, (2) reflecting from a harmonic rejection Cr mirror to cut energies higher than 30 keV, and (3) reflecting from the double-multilayer monochromator (DMM) to select an energy of 24 keV. This energy is in the range of optimal energies (20–30 keV) providing the highest flux at the bending-magnet beamline 2-BM, and gives a high contrast between materials included in the gas-hydrate formation process (see Nikitin *et al.*, 2020 ▸). The resulting beam height for this energy is approximately two times smaller than the detector height. With 2× magnification, we were able to perform measurements with a 4.2 mm × 1.75 mm field of view.

### 3.2. Tomographic imaging

For the experiment we used Ottawa fine white sand (grain sizes 125–250 µm) adding 10% mass fraction of fluid into the pore space. The porosity of the sample was 0.35. Fluid consisted of 5% NaBr solution in water, which improves X-ray contrast between fluid and gas-hydrate during its formation. Chemically pure 2.5-grade methane gas was used for filling pore space and supporting gas-hydrate formation.

Tomographic projections for each scan position were collected in fly scan mode while the sample was continuously rotated at 1.25° per second, yielding 7.5 min for each 360° scan of 3000 projection angles. An additional 2 min after each scan was spent recording dark/flat images, moving the rotation stage vertically, and on camera initialization procedures. The preset time between the beginning of two consecutive 6×-scan vertical measurements was 57 min. In total we collected data for 20 vertical scans over 19 h. All data were stored in 16-bit formatted HDF5 files with a total size of 1.7 Tb.

Acquired projections were stitched and reconstructed by using the ThetaGPU cluster of the Argonne Leadership Computing Facility (ALCF). The reconstruction pipeline consisted of dark/flat-field correction, Paganin phase-retrieval filter, and filtered back-projection implemented via the log-polar-based method on GPUs (Andersson *et al.*, 2016 ▸). In total we recovered 20 sample volumes each having size 6100 × 4896 × 4896 in 32-bit format (10.9 Tb in memory).

**Figure 5:**
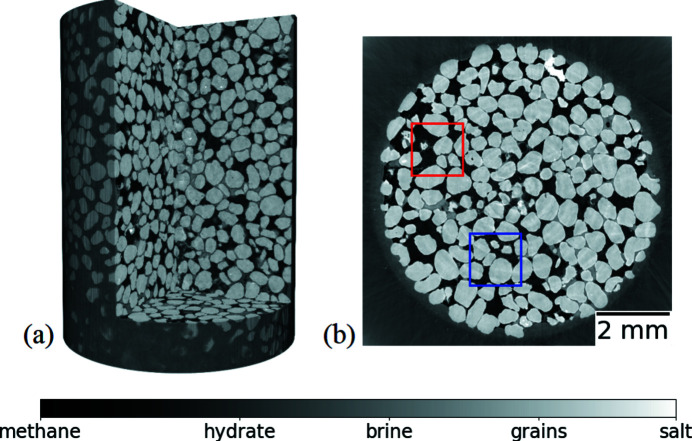
Overview micro-CT image: cut through 3D rendering of the sample (*a*), horizontal slice (*b*).

**Figure 6:**
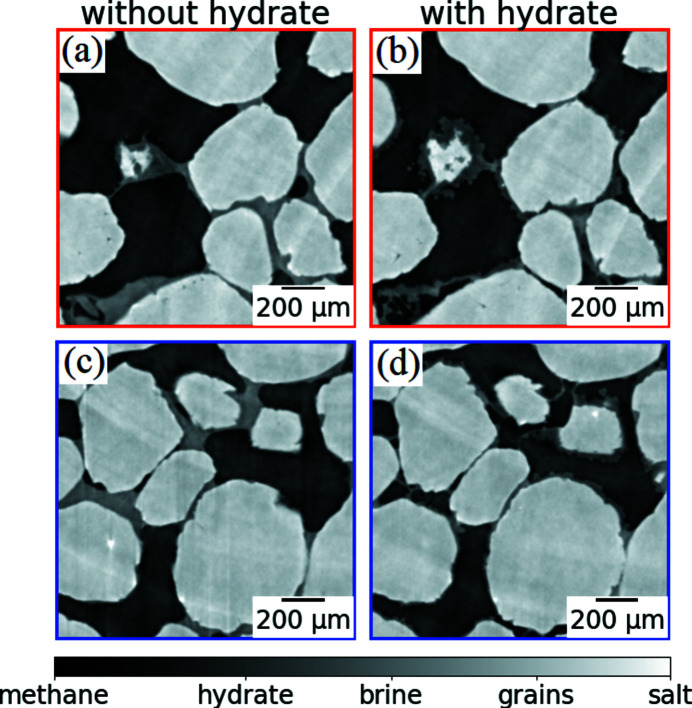
Zoom-in of two regions of interest from Fig. 5 ▸ for two different times: before hydrate formation (*a*, *c*), after hydrate formation (*b*, *d*).

In Fig. 5 ▸ one can see an overview micro-CT image: 3D rendering of the sample (*a*) and horizontal slice (*b*). The gray scale shows different materials as indicated at the bottom of the figure: black – methane gas; dark gray – methane gas hydrate; gray – brine; light gray – sand grains; white – salt. Red and blue square frames highlight areas of interest for detailed imaging, see Fig. 6 ▸.

Fig. 6 ▸ shows zoom-ins of the regions of interest from Fig. 5 ▸: for the red frame (*a*, *b*) and blue frame (*c*, *d*). These images correspond to two times: before the hydrate formation (*a*, *c*) and after the hydrate formation (*b*, *d*). The gray scale shows different materials similarly to the previous figure. In the left column (before the hydrate formation) one can clearly see brine in the pore space forming smooth menisci adjacent to the grains. In the right column (after the hydrate formation) one can see hydrate crystals that envelope the grains and then grow into the pore space. Note that observation of the hydrate-growth morphology is important as it affects acoustic properties and their relation with the hydrate saturation (*cf*. Dugarov *et al.*, 2019 ▸).

**Figure 7:**
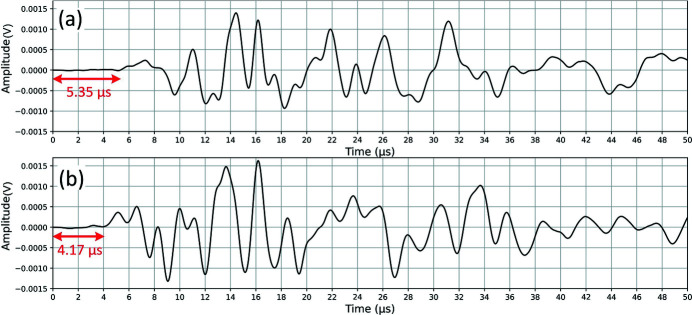
Acoustic traces from the *in situ* experiment at Sector 2-BM: (*a*) initial state, (*b*) state with formed gas hydrates.

Simultaneous acoustic measurements help to follow changes in the mechanical properties during hydrate formation (it may be ice formation, any other phase transition or chemical reaction). In Fig. 7 ▸ we show examples of two acoustic traces recorded by the lower acoustic sensor. They correspond to the two sample states (with and without formed hydrates) shown in Fig. 6 ▸. One can see that the P-wave arrives later [Fig. 7 ▸(*a*)], *i.e.* travels slower through the sample without the gas hydrate [Figs. 6 ▸(*a*) and 6(*c*)]. Later in the experiment the P-wave starts arriving earlier [Fig. 7 ▸(*b*)], *i.e.* travels faster through the sample after the hydrate formation in it [Figs. 6 ▸(*b*) and 6(*d*)]. Thus we obtain a quantitative measure of the material stiffening due to gas-hydrate formation in its pore space.

### 3.3. Acoustic properties

The source impulse from the upper piezo-ceramic acoustic sensor (see Fig. 1 ▸) has the form of a step function with rising time of 0.5 µs, amplitude of 150 V, and repetition rate of 800 kHz. The lower acoustic sensor is connected to an oscilloscope. It is used for recording acoustic waves with a sampling rate of 125 MHz or higher, and a recording length of 50 µs or longer. To improve the signal-to-noise ratio we accumulate 100 signals from repeated generator pulses.

The sample diameter should be comparable with or larger than the wavelength to ensure that we study the body P-wave rather than the pivotal P-wave. For loose sandy materials, variations of P-wave velocity may vary from 1500 to 3500 m s^−1^ (see Duchkov *et al.*, 2019 ▸). Given the signal period of about 2 µs, we can estimate the wave length in the sample as a value between 3 and 7 mm. Thus we decided to make the sample diameter about 8–9 mm which still guarantees that we measure the velocity of the body P-wave.

**Figure 8:**
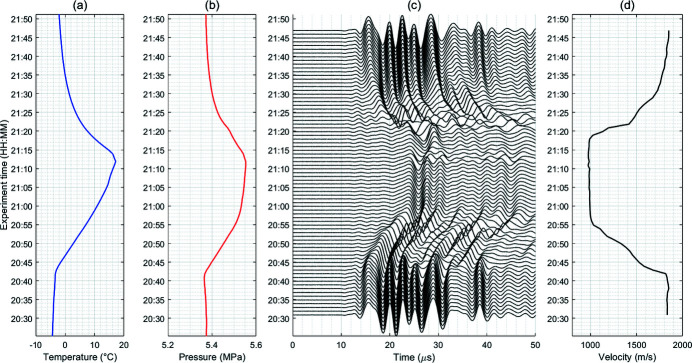
Repeated measurements of temperature (*a*), pore pressure (*b*), acoustic traces (*c*), and derived P-wave velocities (*d*). Measurement time is indicated along the vertical axis; the confining pressure is 8 MPa.

We repeated the measurements every minute and show the data in Fig. 8 ▸. One can see the measurement time along the vertical axis, from 20:30 to 21:50. We show time variations of temperature (*a*), pore pressure (*b*), acoustic traces at different times (*c*), and P-wave velocities derived from these traces (*d*). Each acoustic trace shows arrivals of waveforms propagating from the upper transmitter to the lower receiver. At early times (20:30–20:40) the waves arrive earlier (the first strong maximum roughly at 15 µs). For later measurements (21:00–21:20) the waves arrive later (the first strong maximum at around 24 µs). The P-wave velocity is estimated using a simple formula: *V*_p_ = *l*/(*t*_p_ − *t*_0_), where *l* is the sample length, *t*_p_ is the arrival time of the P-wave detected from the acoustic trace, and *t*_0_ is the sensor delay time (measured separately by connecting two sensors, transmitter and receiver, together).

In order to interpret the acoustic data properly one needs to measure the sample length (17.88 mm in our experiments). We measured the sample length by looking at X-ray images for one projection angle and moving the vertical stage. First, we slid the sample stage up to the border of the upper acoustic sensor. Then we slid the stage down to the border of the bottom sensor. The sample height was measured as the distance between these two marks.

## 4. Discussion and outlook

We have presented the first experimental cell for simultaneous measurement of acoustic properties of a sample and dynamic synchrotron-based micro-tomographic imaging. The cell is designed for *in situ* studying of samples that are 9 mm in diameter, at confining and pore pressures up to 12 MPa, and temperatures down to −20°C. It was successfully tested for measuring changes in acoustic properties of the sample during gas-hydrate formation in its pore space.

The cell is portable and can be easily assembled and operated at different X-ray sources. Additional devices for generating signal impulses and varying the pressure and temperature conditions are typically available upon request at all synchrotron facilities. We have successfully tested the cell at the APS Sector 2-BM operating with filters, mirrors, and DMM for adjusting the beam energy, as well as at the VEPP-3 station where the X-ray beam is regulated only with filters. The experimental hutch at VEPP-3 is also significantly smaller than the one at 2-BM (4 m^2^ versus 30 m^2^) but still allows for appropriate installation of all necessary devices to conduct gas-hydrate formation experiments.

The cell can be used for time-resolved three-dimensional studies of physical/chemical processes in the Earth subsurface: geochemical reactions (for example, in tailings dumps), phase transitions that considerably change the mechanical properties of geological materials (*e.g.* permafrost degradation) and others.

Future plans include improvement of the acoustic sensors to measure velocities of both P- and S-waves. This is important for better characterizing changes in the mechanical properties of a sample. Also, adding Peltier devices for heating the cell will broaden the number of geochemical processes that can be studied in detail. Finally, we plan to consider using new instruments available at Sector 2-BM of the APS. For instance, a new Optique Peter system with automatic remote lens changing allows for high-resolution study of the regions of interest containing gas-hydrates. Streaming data analysis and real-time reconstruction have become recently available at Sector 2-BM of the APS. These new tools may help in understanding the behavior of fast processes such as water redistribution or hydrate dissociation. 

## Supplementary Material

Description of all parts for construction of the environmental cell, technical drawings. DOI: 10.1107/S1600577521013308/ye5011sup1.pdf
