# Supplementary material for: Environmental cell for in situ X-ray synchrotron micro-CT imaging with simultaneous acoustic measurements
Source: J Synchrotron Radiat. 2022 Jan 27;29(Pt 2):515–21. doi: 10.1107/S1600577521013308 (PMC8900851; doi:10.1107/S1600577521013308)
Supplement: Supplementary file 1 [file s-29-00515-sup1.pdf]

JOURNAL OF SYNCHROTRON RADIATION

Volume 29 (2022)

Supporting information for article:

Environmental cell for *in situ* X-ray synchrotron micro-CT imaging with simultaneous acoustic measurements

Arkady N. Drobchik, Viktor V. Nikitin, Mikhail I. Fokin, Geser A. Dugarov, Pavel D. Shevchenko, Alex L. Deriy, Andrey Yu. Manakov, Konstantin E. Kuper and Anton A. Duchkov

Supplementary data.

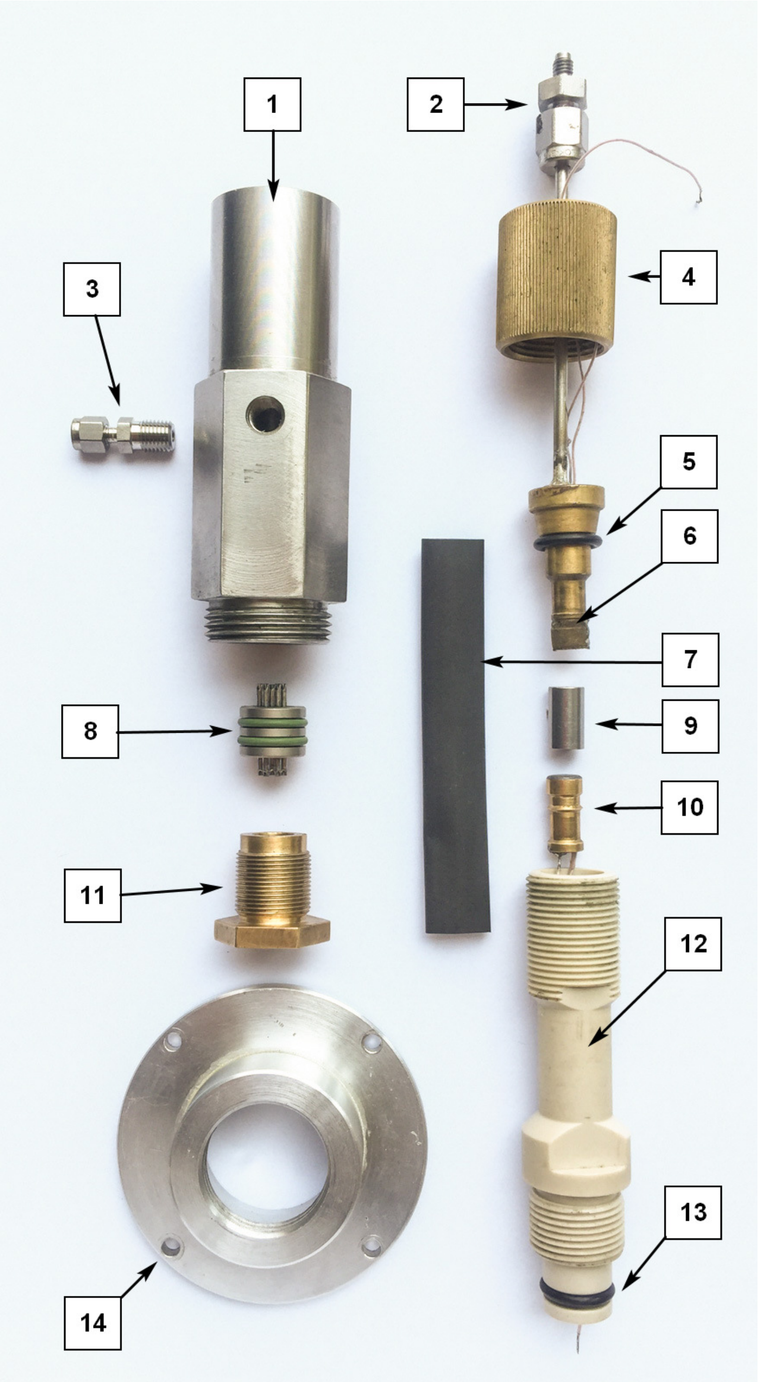

Fig. S1. Appearance of the cell components. The labels are explained in Table S1 below.

Table S1. *Components of the cell from Fig. S1*

№	Description
1.	Stainless body
2.	1/16" - 1/8" connector for tube fitting
3.	Male Connector, 1/16" Tube OD x 1/8" Male ISO Tapered Thread
4.	Brass union nut
5.	Oil-Resistant Hard Buna-N O-Ring 0.4" ID, 0.08" wide
6.	Upper acoustic sensor
7.	Viton heat-shrink tubing 0.38" ID (before shrinking), 1.5" length
8.	High-pressure electrical feedthrough connector
9.	Titanium reference sample 0.4" length
10.	Bottom acoustic sensor
11.	Locking screw with hole
12.	PEEK Tube
13.	Oil-Resistant Hard Buna-N O-Ring 0.5" ID, 0.1" wide
14.	Aluminium base

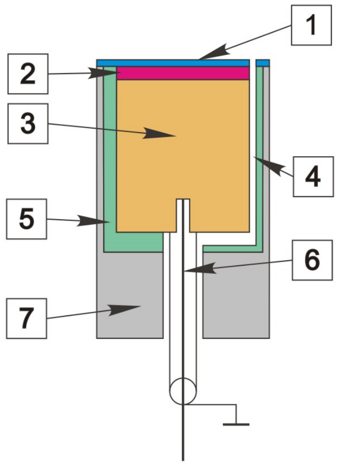

Fig. S2. Scheme of the acoustic sensor. The labels are explained in Table S2 below.

Table S2. *Components of the acoustic sensor from Fig. S2*

№	Description
1.	Cooper cover
2.	Piezoceramic disk, 0.18 x 0.04”
3.	Brass Bekker
4.	Gas supply channel
5.	Dielectric insert with micro channels for gas supply
6.	Coaxial wire
7.	Brass sensor body

Technical drawings

017

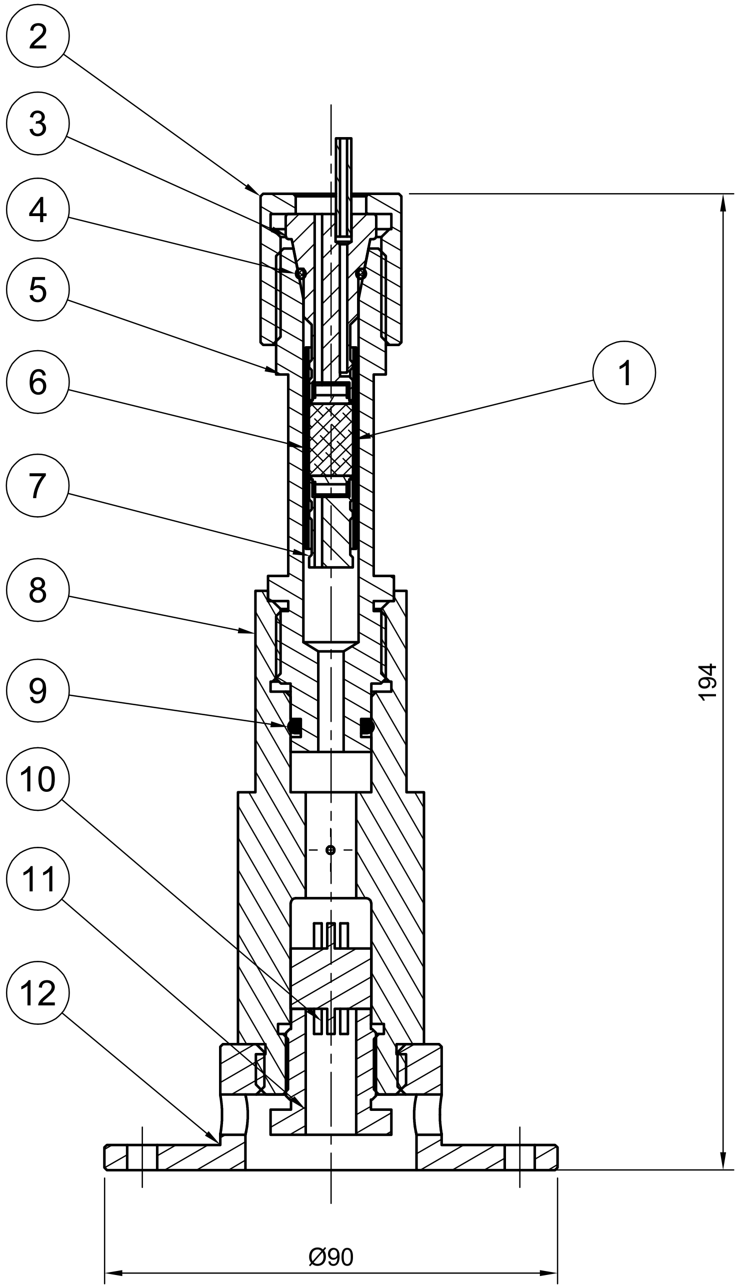

Parts List			
Item	Qty	Part Number	Material
1	1	Sample	
2	1	Top lock nut	Brass
3	1	Top acoustic sensor	
4	1	Oil-Resistant Hard Buna-N O-Ring 0.4” ID, 0.08” wide	Synthetic Rubber
5	1	PEEK tube	PEEK
6	1	Viton heat-shrink tubing 0.38” ID	Viton
7	1	Bottom acoustic sensor	
8	1	Stainless body	Stainless steel
9	1	Oil-Resistant Hard Buna-N O-Ring 0.5” ID, 0.1” wide	Synthetic Rubber
10	1	High-pressure electrical feedthrough connector	
11	1	Bottom lock nut	Brass
12	1	Base	Aluminium

Dept.	Technical reference	Created by Arkadiy Drobchik 11.09.2019	Approved by
		Document type	Document status
		Title Environmental cell	DWG No.
			Rev. / Date of issue / Sheet 1/8

017

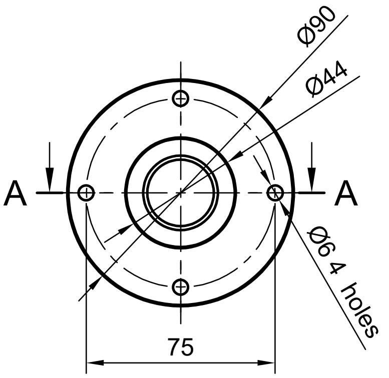

A-A (1:2)

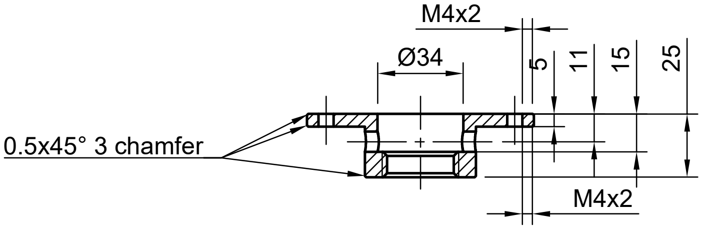

Material: Aluminum D16T
Qty: 1 pc.
Unspecified limit deviations H12, h12

Dept.	Technical reference	Created by<br>Arkadiy Drobchik 11.09.2019	Approved by		
		Document type	Document status		
		Title<br>Base	DWG No.		
			Rev.	Date of issue	Sheet<br>2/8

017

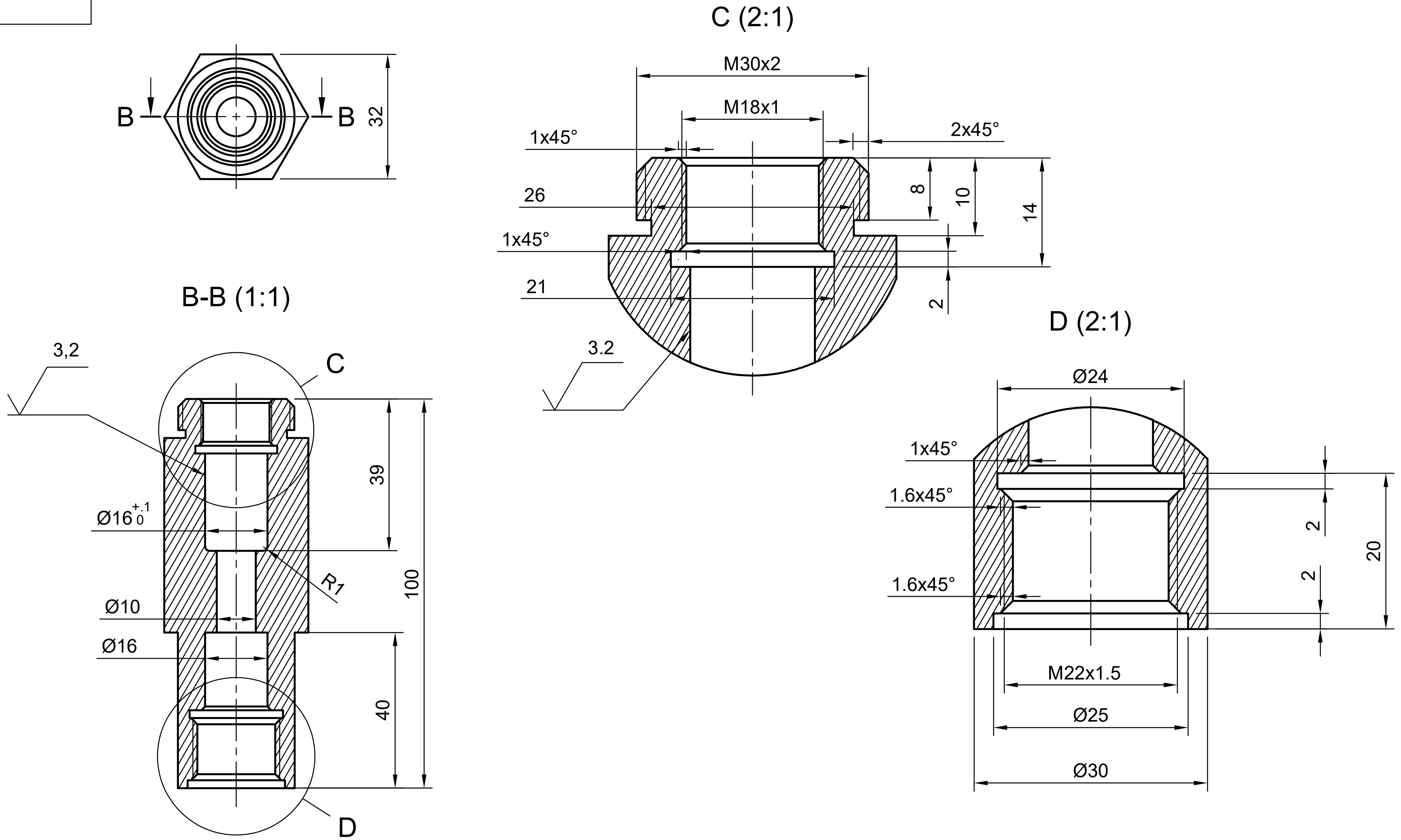

Material: Steel 08X18H10
Qty: 1 pc.
Unspecified limit deviations H12, h12

Dept.	Technical reference	Created by	Approved by
		Arkadiy Drobchik 11.09.2019	
		Document type	Document status
		Title: Stainless body	DWG No.
			Rev. / Date of issue / Sheet 3/8

017

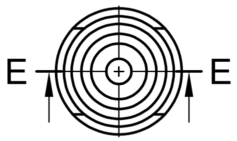

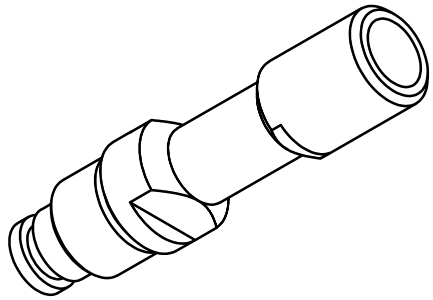

E-E (1:1)

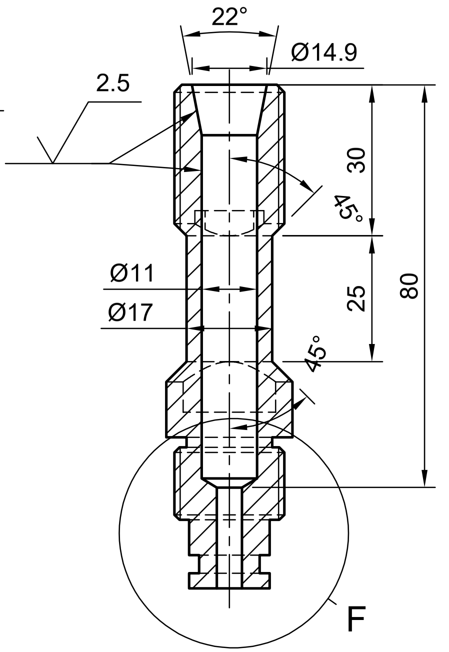

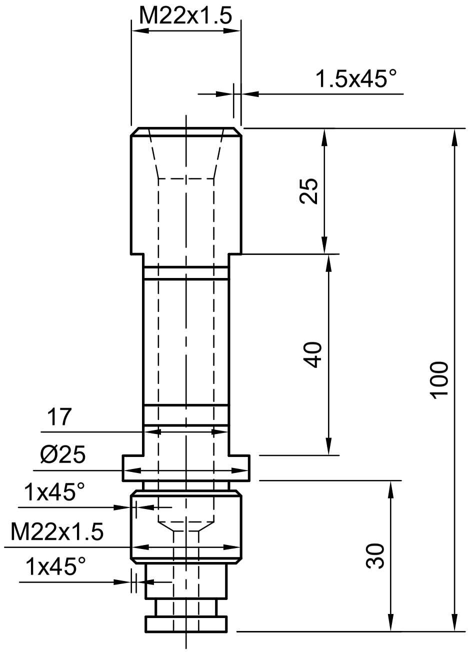

F (2:1)

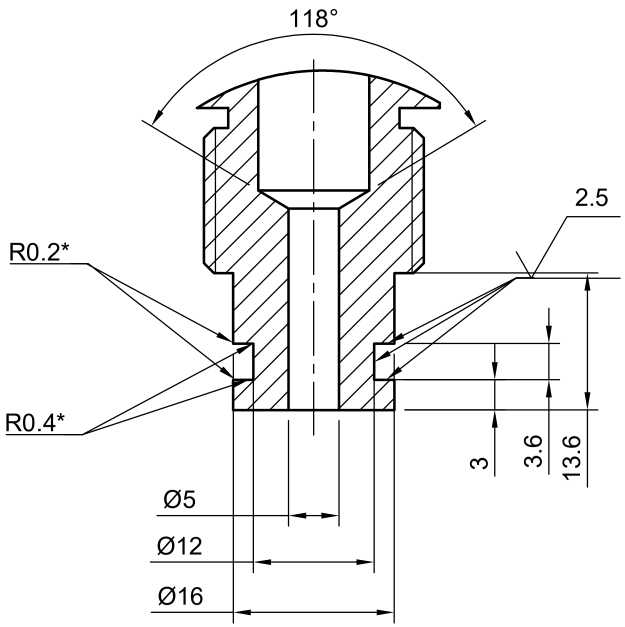

Material: PEEK
Qty: 1 pc.
Unspecified limit deviations H12, h12
* Dimension provided by tool

Dept.	Technical reference	Created by: Arkadiy Drobchik 11.09.2019	Approved by		
		Document type	Document status		
		Title: PEEK tube	DWG No.		
			Rev.	Date of issue	Sheet 4/8

017

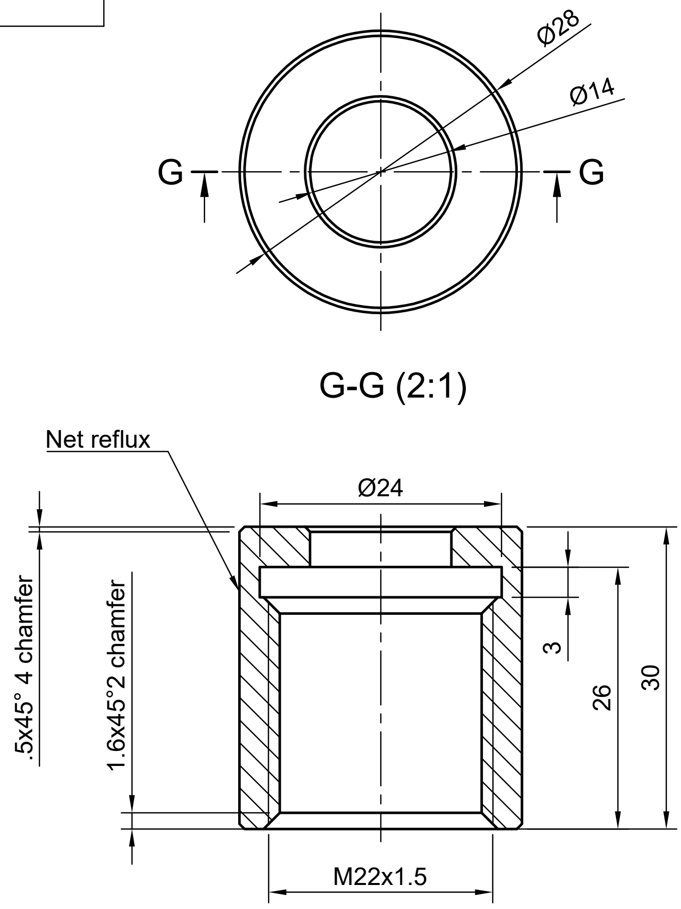

Material: Brass
Qty: 1 pc.
Unspecified limit deviations H12, h12

Dept.	Technical reference	Created by<br>Arkadiy Drobchik 11.09.2019	Approved by
		Document type	Document status
		Title<br>Top lock nut	DWG No.
			Rev. / Date of issue / Sheet 5/8

017

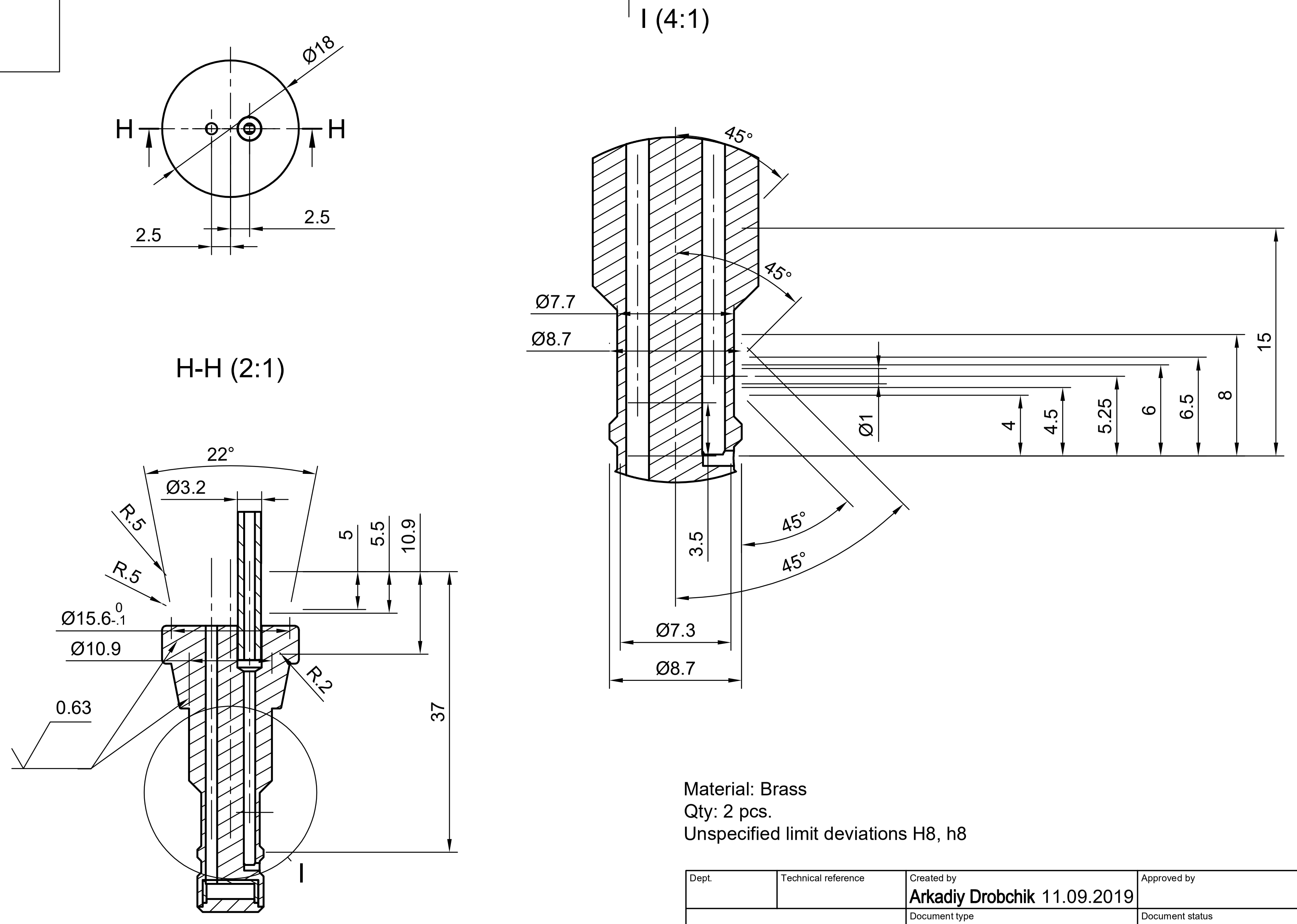

Material: Brass
Qty: 2 pcs.
Unspecified limit deviations H8, h8

Dept.	Technical reference	Created by: Arkadiy Drobchik 11.09.2019	Approved by		
		Document type	Document status		
		Title: **Top acoustic sensor**	DWG No.		
			Rev.	Date of issue	Sheet 6/8

017

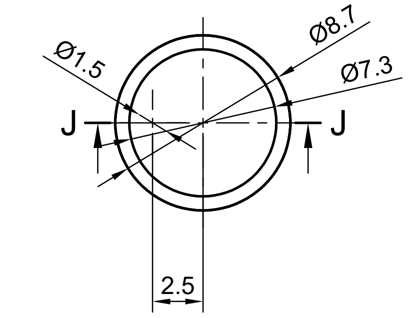

J-J (4:1)

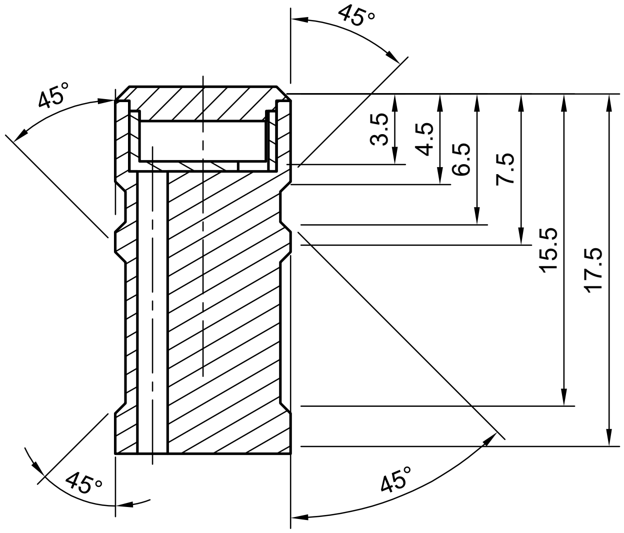

Material: Brass
Qty: 2 pcs.
Unspecified limit deviations H8, h8

Dept.	Technical reference	Created by Arkadiy Drobchik 11.09.2019	Approved by
		Document type	Document status
		Title **Bottom acoustic sensor**	DWG No.
			Rev. | Date of issue | Sheet 7/8

017

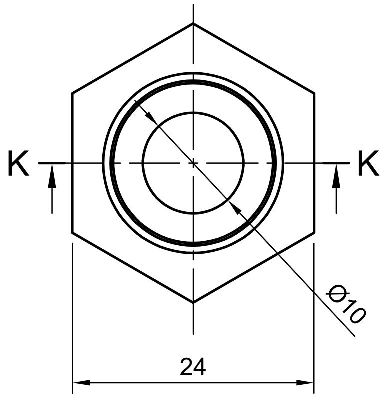

K-K (2:1)

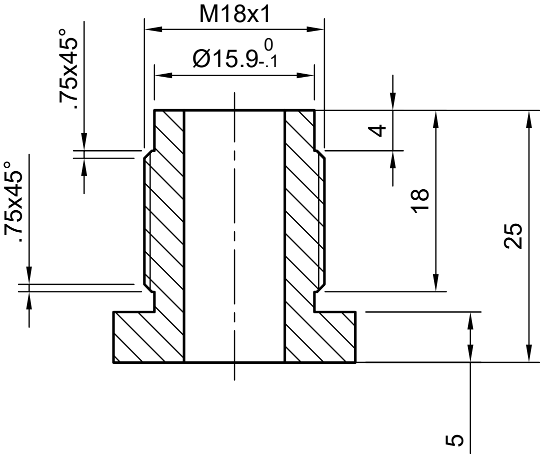

Material: Brass
Qty: 1 pc.
Unspecified limit deviations H10, h10

Dept.	Technical reference	Created by	Approved by
		Arkadiy Drobchik 11.09.2019	
		Document type	Document status
		Title: Bottom lock nut	DWG No.
			Rev. / Date of issue / Sheet 8/8
